# Which behavioral patterns score points in taekwondo matches? An analysis of the Roma 2019 World Grand Prix finalists

**DOI:** 10.3389/fspor.2025.1572945

**Published:** 2025-04-14

**Authors:** Yarisel Quiñones Rodríguez, Gennaro Apollaro, Carmen Fúnez, Emanuela Faelli, Antonio Hernández-Mendo, Verónica Morales-Sánchez, Coral Falcó

**Affiliations:** ^1^ Department of Physical Education and Sports, Faculty of Education and Sport Sciences, University of Granada, Melilla, Spain; ^2^ Department of Neuroscience, Rehabilitation, Ophthalmology, Genetics and Maternal Child Health, University of Genoa, Genoa, Italy; ^3^ Centro Polifunzionale di Scienze Motorie, University of Genoa, Genoa, Italy; ^4^ Consejería de Desarrollo Educativo y Formación Profesional, Junta de Andalucía, Seville, Spain; ^5^ Department of Experimental Medicine, Section of Human Physiology, University of Genoa, Genoa, Italy; ^6^ Department of Social Psychology, Social Work, Social Services and Social Anthropology, University of Málaga, Málaga, Spain; ^7^ Department of Sport, Food and Natural Sciences, Western Norway University of Applied Sciences, Bergen, Norway

**Keywords:** taekwondo, observational methodology, polar coordinate analysis, function estimation, sequential analysis, technical-tactical patterns

## Abstract

**Introduction:**

Observational methodology facilitates the study of situational factors in taekwondo matches, examines observable technical-tactical behaviors, and reveals various relationships, thus generating behavioral patterns. The aim of this study was to examine the technical-tactical scoring actions in the final matches of the Roma 2019 World Taekwondo Grand Prix, using polar coordinate analysis and function estimation.

**Methods:**

A point/nomothetic/multidimensional (P/N/M) observational design was applied, where the movements of all athletes were analyzed in relation to their behaviors and dimensions that align with the criteria of the observation instrument. Seven matches were observed (female, *n* = 3; male, *n* = 4) for a total of 1,382 registrations. The observation tool used for this study was designed “*ad hoc*” using the HOISAN tool. It consisted of five criteria (tactical action, technique type, strike area, sidedness, and scoring) and 30 categories. The tool was designed by three observers. Pearson's, Spearman's, and Kendall's tau-b interobserver and intraobserver correlation coefficients were estimated. Five focal categories related to the criterion “scoring” were considered: one point, two points, three points, four points, and five points. Relationships with a vector length ≥1.96 were considered significant (*p* < 0.05). A function estimation analysis was performed to validate the observed values (using linear, polynomial, exponential, and logarithmic regression, and polynomial interpolation) using Estimación de Funciones software.

**Results and discussion:**

The results allow for the development of new training and competition strategies based on the different technical-tactical actions that establish a significant pattern in the realization of a scoring action. Specifically, one point was obtained through a direct attack, a correction, or a subsequent counterattack. Two points were obtained through a simultaneous counterattack, while three points were obtained through an anticipation or a melee tactical action. Four points and five points were obtained through a direct attack or a simultaneous counterattack. Two points and three points were obtained with the left leg, while four points were obtained with the right leg. Moreover, three points and four points inhibited the right and left side of the body, respectively. Linear techniques were also inhibited when obtaining one point and five points, while circular techniques were used before or after one, two, and five points, showing that the circular technique was the most used.

## 1 Introduction

Taekwondo is one of the most widely followed and practiced combat sports worldwide with 213 National Member Associations of World Taekwondo (WT) (1). Taekwondo's level of notoriety is well evidenced by the unveiling in 2023 of a taekwondo statue at the Olympic Museum in Lausanne, close to both the Olympic Flame and the statue of the founder of the Olympic Movement (2). At the same time, its notoriety goes hand in hand with constant rule changes to ensure the matches are dynamic and spectacular (3). In this regard, the current scoring system provides a specific score ranging from one to five points based on the type of attack made: one point for a valid punch to the torso or for each warning, two points for a valid kick to the torso, three points for a valid kick to the head, four points for a valid spin kick to the torso, and five points for a valid spin kick to the head (4).

To date, numerous studies have used observational methodology to analyze the behavior of taekwondo athletes during international competitions, with the aim of anticipating, improving, and developing new technical-tactical actions (5–15). Menescardi et al. (12) analyzed the technical-tactical actions of taekwondo athletes in the London 2012 Olympic Games (OG) by weight category and match outcome for each sex. This layering of the analysis revealed distinct technical-tactical behaviors as lighter athletes performed more actions than heavier athletes. Heavier female athletes performed more defensive actions than lighter athletes. Winners performed more anticipated one- and three-point actions than losers. Furthermore, Menescardi et al. (15) studied the effectiveness of technical-tactical actions during the London 2012 OG according to athletes’ sex. They found differences in the use of tactics, techniques, zones, legs, and guards to score points. Among the main results, circular techniques were the most effective for scoring one point, linear for scoring three points, and spin techniques for scoring two and four points. The rear leg was more effective for scoring one, two, and four points. Observational methodology facilitates the study of situational factors in taekwondo matches, examines observable technical and tactical behaviors, and reveals various relationships (such as frequency, order, and timing), thus generating behavioral patterns (9). Since every movement or action can be crucial in taekwondo combat, the use of observational methodology is essential, and the collected data enables coaches to plan and devise strategies for future competitions (8). However, Gutiérrez-Santiago et al. (16) highlighted that the problem with the majority of these types of investigations is that they are mainly descriptive studies, analyzing the frequency of movements, without providing a sequential study.

In this sense, in most studies that employed observational methodology in taekwondo over the past decade, one of the analytical techniques implemented was polar coordinate analysis. Through this analysis, the relationships between a focal behavior and the conditional behaviors within the taxonomic system are estimated, based on the sequential analysis of prospective and retrospective lags (17). Simultaneously, the “genuine technique” accounts for negative lags and detects the consistency of previous actions of order *n* with the criterion behavior, integrating both prospective and retrospective perspectives (18). This technique employs the Zsum (Zsum = ∑*z*/√*n*) parameter and the values obtained represent a vector representation of the different taxonomic categories (8, 19, 20). The behavioral map with polar coordinates illustrates, in the form of vectors, the longitude and angle values, which convey information about strategies or techniques with varying levels of success (8, 18). To date, polar coordinate analysis is widely employed in the scientific literature, with taekwondo being one of the leading sports in its application (3, 8, 9, 13, 14, 21–24). For example, this observational methodology has been used to analyze the behavioral patterns used to score points in the London 2012 OG based on sex and weight category (25), to analyze the technical-tactical actions performed by athletes in the Rio 2016 OG based on the outcome of the match (14), or to explore the effective patterns associated with the scoring of two medalists in the 2012 and 2016 OGs (11). Recently, Gamero-Castillero et al. (3) used polar coordinate analysis to identify the technical-tactical actions with which winning and losing athletes scored in the finals of the Roma 2019 World Taekwondo Grand Prix Series-1 (WGPS). Among the winners, obtaining one point was preceded and followed by direct attack and correction behaviors, with a single or double technique, using a circular kick to the head. Among the losers, obtaining one point was preceded and followed by a subsequent counterattack, with a circular kick executed with the right leg. Winners and losers showed a similar behavioral pattern when scoring two points, since both groups used a punch to obtain a point, while the opponent received a penalty at the same time. Winners showed melee, punch, and head strike behaviors, while losers performed circular actions to score three points. Four and five points were scored only by the winners with a valid turning kick to the trunk protector and a valid kick to the helmet preceded by a turn, respectively.

It is interesting to highlight that in the study of Gutiérrez-Santiago et al. (16), only four common analytical techniques in observational methodology (i.e., traditional statistical analysis, T-pattern detection, sequential lag analysis, and polar coordinate analysis) were used in parallel to analyze international male competitions. According to the authors, the usefulness of this combination is that the results obtained through one analytical technique are corroborated by the others and, overall, provide a broader and deeper insight for coaches and athletes. In this context, function estimation, through the use of regression models (linear, polynomial, logarithmic, and exponential) and polynomial interpolation, has never been combined with other analytical techniques in the competitive context of taekwondo, despite its potential for predicting unobserved data and validating observed data (23). Therefore, the combination of polar coordinate analysis and function estimation could offer a more comprehensive assessment of technical-tactical behavior in taekwondo matches. In this sense, the aim of the present study was to examine the technical-tactical scoring actions in both male and female matches at the Roma 2019 WGPS-1 finals using polar coordinate analysis and function estimation. The WGPS is a closed-numbered event introduced in 2013, in which the top 31 athletes in the world ranking and one athlete from the host country of the edition per weight category participate (26). The finals of this international competition were specifically chosen to expand the understanding of the recent results of Gamero-Castillero et al. (3) through a combination of analysis and evaluation techniques never before used in taekwondo.

## 2 Materials and methods

### 2.1 Experimental design

A point/nomothetic/multidimensional (P/N/M) observational design, situated in quadrant III of observational designs, was used, where the movements of all athletes are analyzed in relation to their behaviors and dimensions that align with the criteria of the observation instrument (27).

### 2.2 Participants

The sample consists of four male and three female matches from the finals of the Roma 2019 WGPS-1 (7–9 June 2019). One female match was not held due to an injury to one athlete. The finals in the male category were as follows: flyweight ≤58 kg (South Korea vs. Spain), featherweight ≤68 kg (Iran vs. South Korea), welterweight ≤80 kg (Russia vs. Spain), and heavyweight ≥80 kg (Russia vs. Kazakhstan). The finals in the female category were as follows: flyweight ≤49 kg (Russia vs. South Korea), featherweight ≤57 kg (South Korea vs. Turkey), and welterweight ≤67 kg (Croatia vs. South Korea). In total, 695 records were obtained from the male matches and 687 from the female matches, with 1,382 technical-tactical actions recorded in total. The data necessary for generalization were obtained from the study by Gamero-Castillero et al. (3) using a generalizability analysis to unify the definitions of reliability, validity, and accuracy (28). All the matches included in this study were recorded from the open-source website (https://www.youtube.com), and no approval by an Ethical Committee was required, as the study did not involve any experimentation, in accordance with the rules laid down by the Belmont Report (29).

### 2.3 Measures

The observation tool used for this study was designed “*ad hoc*” by Gamero-Castillero et al. (3) using HOISAN v.1.5.6 software (30, 31). It is a mixed system of field formats and a system of exhaustive and mutually exclusive (E/ME) categories in each criterion (20, 32, 33). It consists of five criteria (tactical action, technique type, strike area, sidedness, and scoring) and 30 categories. Each category has its own coding system and categorical core (Table 1). Technical-tactical actions and sports rules were considered in the selection of criteria and categories (8, 34, 35).

**Table 1:** Criteria, categories, codes, and categorical core of the observational tool.

Criteria	Categories	Code	Categorical core or description
Tactical action	Direct attack	DA	Offensive move not preceded by any prior movement and performed with the intention of scoring.
	Indirect attack	IA	Offensive move preceded by a prior movement (change of position, jump, etc.) and performed with the intention of scoring.
	Correction	CORR	Move combining two technical actions executed with the same leg or different legs.
	Melee	MEL	Attacking action in which the opponent is at very close range.
	Subsequent counterattack	SUBCA	Defensive move initiated after the opponent's attacking action.
	Simultaneous counterattack	SIMCA	Defensive move initiated at the same time as the opponent’s attacking action.
	Anticipatory counterattack	ANTCA	Defensive move that anticipates the attacking action initiated by the opponent.
	Clash	CLA	Defensive action attempting to impede the opponent's attack using the legs.
	Block	BLO	Move performed with the hands, used when the attack cannot be dodged.
	Dodge	DOD	Backward movement to evade the opponent's attack.
Type of technique	Linear	LIN	Attack executed with a straight kick.
	Circular	CIR	Attack executed with a kick involving a circular movement of the hip or knee.
	Turning	TUR	Attack executed with a 180° or 360° turn.
	Punch	PUN	Strike delivered with the hand closed into a fist.
	Simple	SING	Corrective action, performed with the same leg, following an up-down or down-up trajectory.
	Double	DOU	Corrective action, performed with different legs.
	None	NOT	No technique executed.
Strike area	Head	HEA	Strike to the permitted areas of the head. In the case of a single or double correction, the head will be considered the last action.
	Trunk	TRU	Strike to the permitted areas of the trunk. In the case of a single or double correction, the trunk will be considered the last action.
	None	NOA	No strike area.
Sidedness	Right	RT	Technique executed from the right side of the body.
	Left	LF	Technique executed from the left side of the body.
	Neutral	NEU	Kick or punch executed from an imperceptible angle of view, also present in dodging and blocking.
Scoring	Zero points	0P	Ineffective attack with the first or foot.
	One point	1P	Valid punch to the trunk.
	Two points	2P	Valid kick to the trunk.
	Three points	3P	Valid kick to the head.
	Four points	4P	Valid turning kick to the trunk or achieving a correction to the trunk only.
	Five points	5P	Valid turning kick to the head or achieving a correction to the trunk and head.
	Gam-jeon	GJ	Action entailing a penalty or warning (crossing the boundary line, falling down, avoiding the match, attacking the fallen opponent, etc.) and signaled by the referee with a horizontal movement of the arm. This action gives one point to the opponent.

### 2.4 Procedures

The tool was designed by three observers under the supervision of an observer experienced in taekwondo and observational methodology. During the training period, observers defined the design, sampling plan, instrument construction, coding system, and recording procedures (36). In addition, a consensus agreement was reached prior to any observations (36, 37). All combat actions involving contact or a dodge to avoid the opponent's attack were considered in the design. As reported in the study by Gamero-Castillero et al. (3), the three observers validated the tool by observing and recording the flyweight ≤58 kg (South Korea vs. Spain) match in the male category and the welterweight ≤67 kg (Croatia vs. South Korea) match in the female category to establish intra/interobserver reliability. The final process involved the first observer, who observed and recorded the bouts analyzed in the polar coordinate analysis.

### 2.5 Statistical analysis

Pearson's, Spearman's, and Kendall's tau-b correlation coefficients (Table 2), and Cohen's Kappa concordance index (Table 3) were estimated using HOISAN v.1.5.6 software (30, 31). The obtained values were considered adequate results for obtaining reliable records.

**Table 2:** Pearson's, Spearman's, and Kendall's tau-b interobserver and intraobserver correlation coefficients.

Coefficient	Interobserver agreement			Intraobserver agreement
	One and two	One and three	Two and three	
Pearson	0.99	0.99	0.99	0.99
Spearman	0.99	0.97	0.99	0.99
Kendall tau-b	0.96	0.94	0.98	0.98

**Table 3:** Cohen's kappa interobserver and intraobserver concordance indexes.

Criteria	Interobserver agreement			Intraobserver agreement
	One and two	One and three	Two and three	
Complete session	0.93	0.91	0.97	0.99
Tactical action	0.92	0.89	0.96	0.97
Type of technique	0.81	0.72	0.86	1
Strike area	0.87	0.87	1	1
Sidedness	0.85	0.85	1	1
Scoring	0.69	0.69	1	1

A generalizability analysis was then performed following the methodology of Blanco-Villaseñor et al. (28) using SAGT v.1.0 software (38). The Category (C) and Observer (O) facets were used, and consequently, the C/O and O/C models were applied. Gamero-Castillero et al. (3) tested the reliability of the observers and found that five matches yielded both a relative and absolute generalizability coefficient of 0.98.

Subsequently, sequential and polar coordinate analyses were performed using HOISAN v.1.5.6 software (30, 31). Five focal categories related to the criterion “scoring” were considered: one point (1P), two points (2P), three points (3P), four points (4P), and five points (5P). Relationships with a vector length ≥1.96 were considered statistically significant (*p* < 0.05). To optimize the graphical representation of the significant vectors obtained in the polar coordinate analysis, the algorithm developed in RStudio software (RStudio, PBC, Boston, MA) by Rodríguez-Medina et al. (39) was used.

The characterization of each quadrant was defined as follows (20):

• Quadrant I (0°–90°): behaviors with a mutually excitatory relationship between the focal and conditioned behaviors in a prospective perspective and a retrospective perspective (+, +).
• Quadrant II (90°–180°): inhibitory relationships in a prospective perspective and excitatory relationships in a retrospective perspective (−, +).
• Quadrant III (180°–270°): inhibitory relationships in prospective and retrospective perspectives (−, −).
• Quadrant IV (270°–360°): inhibition relationships in a retrospective perspective and activation relationships in a prospective perspective (+, −).

Finally, a function estimation analysis was conducted using the Estimación de Funciones v.1.1, developed on the.NET platform (40). The function interpolation software offers approximation methods for this purpose, including linear, polynomial, exponential, and logarithmic regression, as well as polynomial interpolation.

## 3 Results

The results of the polar coordinate analysis and function estimation of the focal behaviors 1P, 2P, 3P, 4P, and 5P, as well as the resulting behavioral maps, are presented below.

### 3.1 1P focal behavior

In quadrant I, the 1P behavior was associated with mutual excitation to a direct attack (DA), correction (CORR), and subsequent counterattack (SUBCA), and with circular (CIR), simple (SING), and double (DOU) techniques. In quadrant III, mutual inhibition occurred with the conditional behaviors indirect attack (IA) and linear(LIN) techniques. Quadrant IV showed an excitation relationship in the retrospective perspective and inhibition in the prospective perspective with the conditional behavior simultaneous counterattack (SIMCA) (Table 4).

**Table 4:** Results of the polar coordinate analysis for the one-point (1P) focal behavior.

Category	Quadrant	ProspP	RetrospP	Radius	Angle
Direct attack (DA)	I	2	1.81	2.69^a^	42.15
Indirect attack (IA)	III	−2	−0.23	2.01^a^	186.70
Correction (CORR)	I	2.82	0.74	2.91^a^	14.82
Subsequent counterattack (SUBCA)	I	2.28	1.32	2.63^a^	29.97
Simultaneous counterattack (SIMCA)	IV	0.71	−1.94	2.07^a^	290.17
Linear (LIN)	III	−0.63	−2.06	2.15^a^	253.10
Circular (CIR)	I	0.71	3.33	3.41^a^	78.05
Simple (SING)	I	2.53	0.49	2.57^a^	10.90
Double (DOU)	I	2.71	1.11	2.92^a^	22.24

^a^ Significant vectors (radius >1.96).

**Figure 1:**
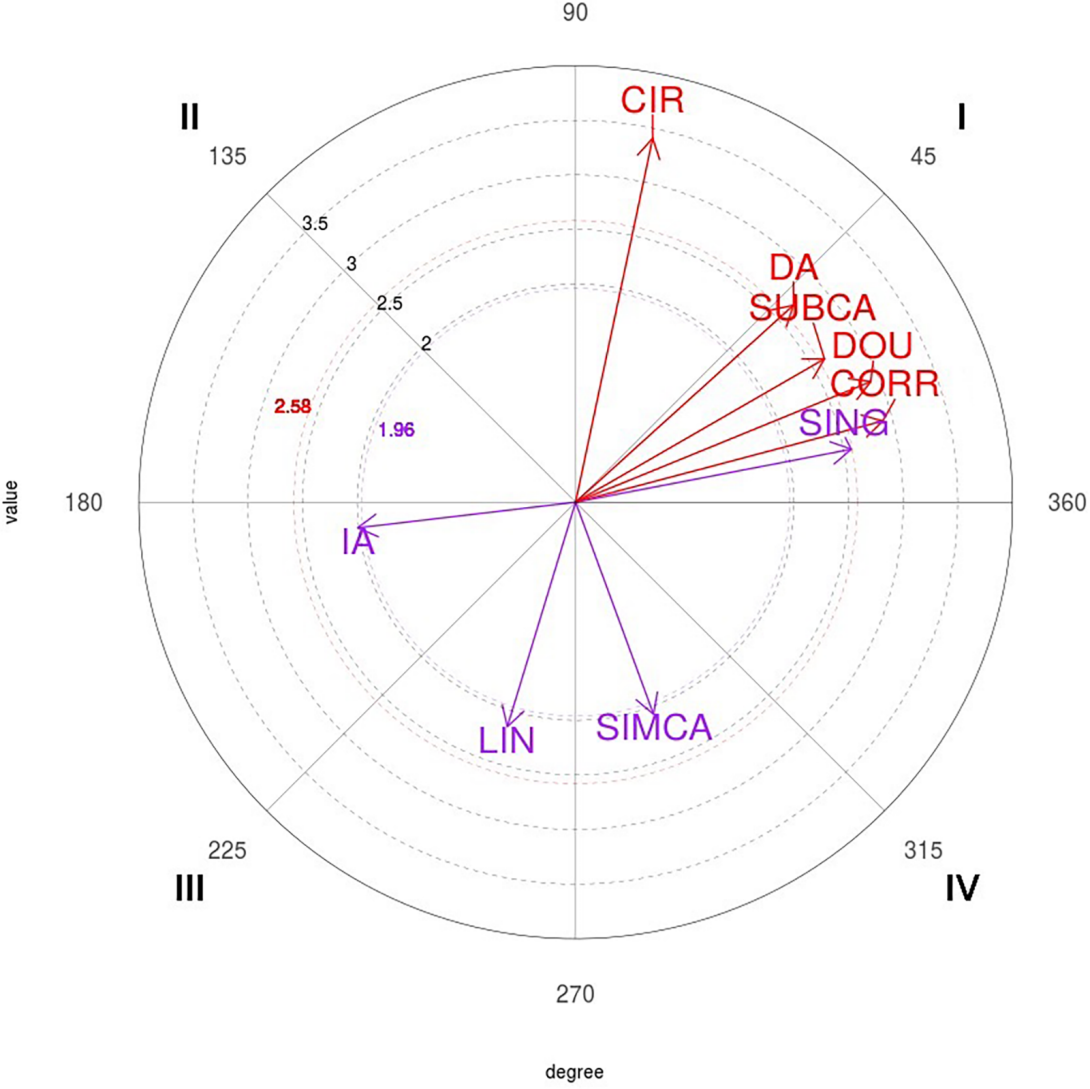
Representation of the behavioral map for the one-point (1P) focal behavior. CIR, circular; DA, direct attack; SUBCA, subsequent counterattack; DOU, double; CORR, correction; SING, simple; SIMCA, simultaneous counterattack; LIN, linear; IA, indirect attack.

The polar coordinate map of the interactions between the 1P focal behavior and conditional behaviors is shown in Figure 1.

The results of function estimation for quadrants I and III associated with focal behavior 1P are presented below. Quadrants II and IV do not allow for model analysis, as they have only one significant relationship in the polar coordinate analysis.

In quadrant I, the results of the function estimation show that polynomial interpolation is the best, with near-zero errors on the order of 10^−14^ and 10^−15^ and perfect correlation. The linear and polynomial regressions provide similar results in terms of errors and explained variance. However, the exponential and logarithmic regressions have a higher relative error, but they are still fairly good models, explaining more than 90% of the variance (Table 5).

**Table 5:** Estimation of quadrant I functions for the one-point (1P) focal behavior.

Formal definition	Approximation method	Absolute error	Relative error	Correlation between *X* and *Y*	Explained variance between *X* and *Y*	Correlation between estimated *X* and *Y*	Explained variance between estimated *X* and *Y*	Correlation estimated *Y* vs. *Y*	Explained variance estimated *Y* vs. *Y*	Function color	Graphic
*f*(*x*) = 4.215 + ×* −1.264	Linear regression	1.0840	0.1806	−0.9605	0.9226	−1	1	0.9605	0.9226	Black	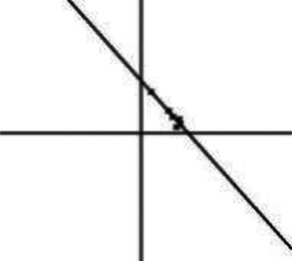
*f*(*x*) = (0.093 * (*x*^2^)) + (−1.586 * (*x*^1^)) + (4.429)	Polynomial regression	1.0842	0.1807	−0.9605	0.9226	−0.9991	0.9982	0.9613	0.9242	Red	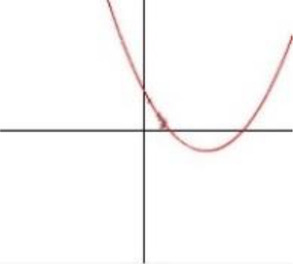
*f*(*x*) = 6.056 * (0.478*^x^*)	Exponential regression	1.6290	0.2715	−0.9605	0.9226	−0.9836	0.9675	0.9504	0.9032	Blue	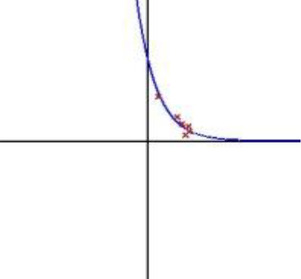
*f*(*x*) = −1.872 * Log(*x*) + (2.757)	Logarithmic regression	1.3491	0.2248	−0.9605	0.9226	−0.9885	0.9771	0.9536	0.9094	Green	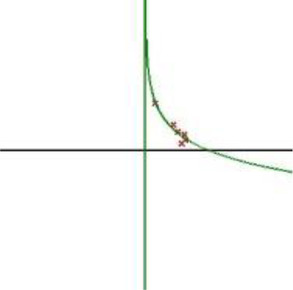
*f*(*x*) = 3.33–1.1781 * (*x* − 0.71) − 0.364 * (*x* − 0.71) * (*x* − 2) − 1.428* (*x* − 0.71) * (*x* − 2) *	Polynomial interpolation	2.11 × 10^−14^	3.52 × 10^−15^	−0.9605	0.9226	−0.9605	0.9226	1	1	Brown	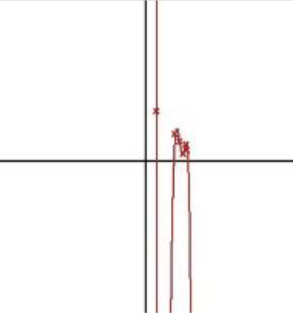
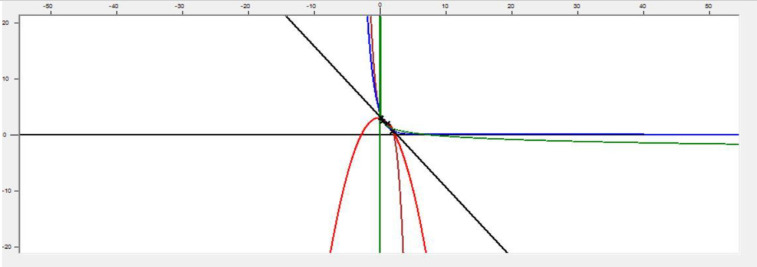											

In quadrant III, all models exhibit a perfect fit to the data, with correlations and explained variances reaching 100%. The errors (absolute and relative) are extremely low, confirming the model's precise alignment with the observed data. The perfect negative correlation of −1 and the 100% explained variance (1) in polynomial interpolation indicate that this model fully accounts for the variability of *Y* (Supplementary Table S1).

### 3.2 2P focal behavior

In quadrant I, the 2P behavior was mutually excitatory with SIMCA, CIR, trunk (TRU), and left (LF). In quadrant II, the behavior exhibited a unique relationship of inhibition in the prospective perspective and excitation in the retrospective perspective with punch (PUN). In quadrant III, reciprocal inhibition emerged with neutral (NEU), none (NOT), and none (NOA). Finally, quadrant IV showed two significant relationships: excitation in the retrospective perspective and inhibition in the prospective perspective with block (BLO) and LIN (Table 6).

**Table 6:** Results of the polar coordinate analysis for the two-point (2P) focal behavior.

Category	Quadrant	ProspP	RetrospP	Radius	Angle
Simultaneous counterattack (SIMCA)	I	1.94	0.57	2.02^a^	16.48
Block (BLO)	IV	0.28	−2.17	2.19^a^	277.38
Linear (LIN)	IV	0.94	−1.95	2.16^a^	295.73
Circular (CIR)	I	0.16	3.02	3.03^a^	87.00
Punch (PUN)	II	−0.02	3.57	3.57^a^	90.37
Trunk (TRU)	I	0.43	2.47	2.51^a^	80.08
Left (LF)	I	1.15	2.10	2.39^a^	61.16
Neutral (NEU)	III	−0.86	−2.53	2.67^a^	251.33
None (NOT)	III	−0.75	−2.44	2.56^a^	252.87
None (NOA)	III	−0.69	−2.40	2.49^a^	253.83

^a^ Significant vectors (radius >1.96).

**Figure 2:**
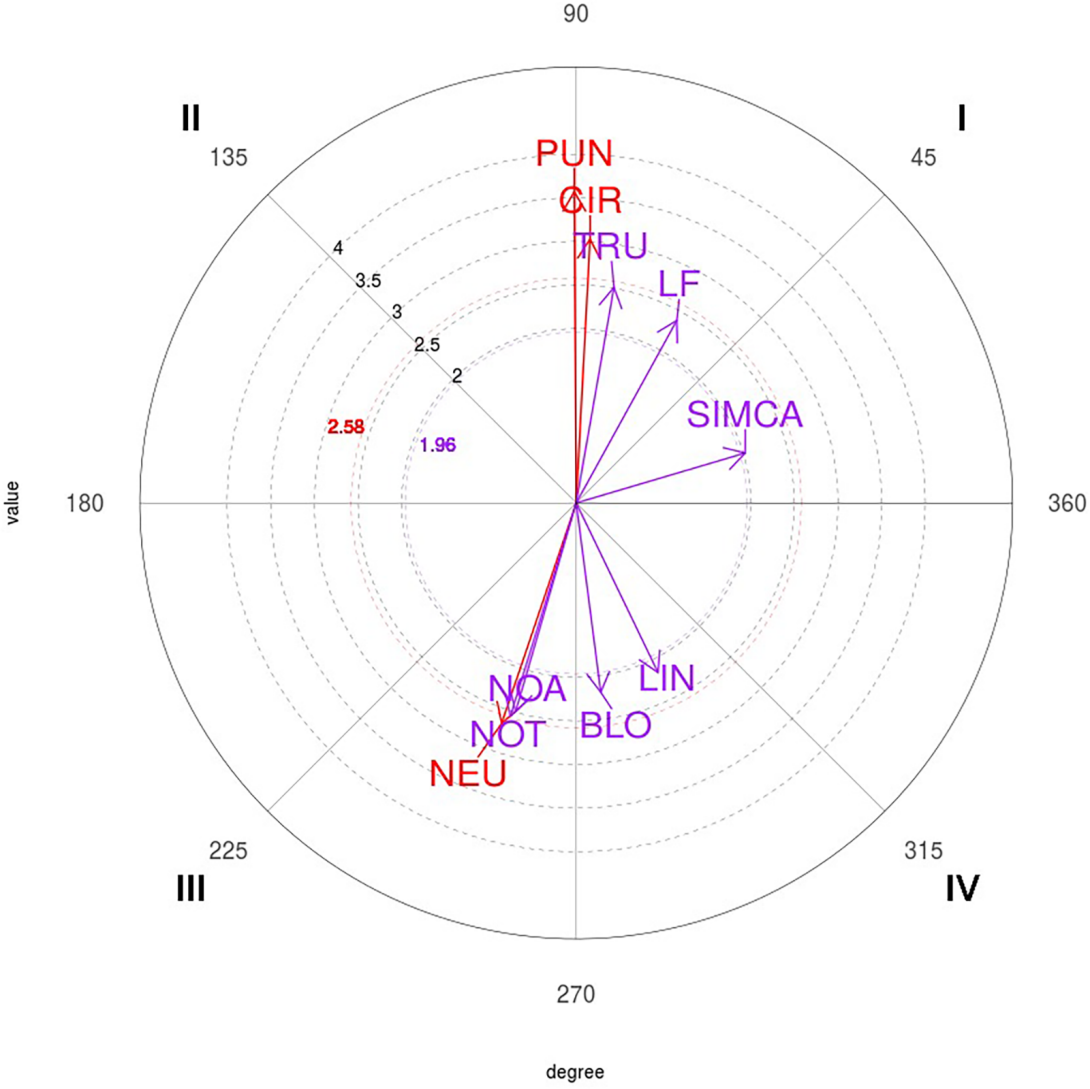
Representation of the behavioral map for the two-point (2P) focal behavior. PUN, punch; CIR, circular; TRU, trunk; LF, left; SIMCA, simultaneous counterattack; LIN, linear; BLO, block; NOA, none; NOT, none; NEU, neutral.

The polar coordinate map of the interactions between 2P focal behavior and conditional behaviors is shown in Figure 2.

The results of the function estimation for 2P focal behavior are presented in Supplementary Tables S2–S4.

In quadrant I, the polynomial interpolation model provided the best fit, with extremely low errors (10^−15^) and a perfect fit across all metrics. Linear and polynomial regression models also showed good fits, while exponential and logarithmic regressions demonstrated lower precision. The high explained variance (94.21%) indicates that this model effectively captures the relationship between behavior and the factors stimulating the 2P score (Supplementary Table S2).

Quadrant III shows an almost perfect fit to the data, with extremely low errors and an explained variance close to 100%. In particular, polynomial interpolation has the lowest errors (10^−16^) and the most accurate fit. The linear and polynomial models also performed exceptionally well, with minimal variance in errors. The data confirm that all three models (linear, polynomial, and polynomial interpolation) provide excellent fits with only minor differences in accuracy (Supplementary Table S3).

Quadrant IV shows that all models fit the data well, with absolute and relative errors being remarkably low and an explained variance of 100%. Differences between models were minimal, suggesting that all methods (linear, polynomial, and logarithmic interpolation) were highly effective. The high accuracy of these models indicates that they correctly represent the data within this specific set (Supplementary Table S4).

### 3.3 3P focal behavior

In quadrant I, the 3P behavior was mutually excitatory with melee (MEL), PUN, head (HEA), and LF. In quadrant II, only one relationship was identified, specifically with anticipatory counterattack (ANTCA). Quadrant III exhibited a single significant relationship with right (RT), while quadrant IV showed a significant association with CIR (Table 7).

**Table 7:** Results of the polar coordinate analysis for the three-point (3P) focal behavior.

Category	Quadrant	ProspP	RetrospP	Radius	Angle
Melee (MEL)	I	1.86	2.62	3.21^a^	54.53
Anticipatory counterattack (ANTCA)	II	−0.78	1.82	1.98^a^	113.27
Circular (CIR)	IV	3.02	−0.25	3.03^a^	355.23
Punch (PUN)	I	2.03	2.03	2.87^a^	45.02
Head (HEA)	I	1.71	2.66	3.16^a^	57.30
Right (RT)	III	−0.21	−2.16	2.17^a^	264.39
Left (LF)	I	1.39	2.33	2.72^a^	59.22

^a^ Significant vectors (radius >1.96).

**Figure 3:**
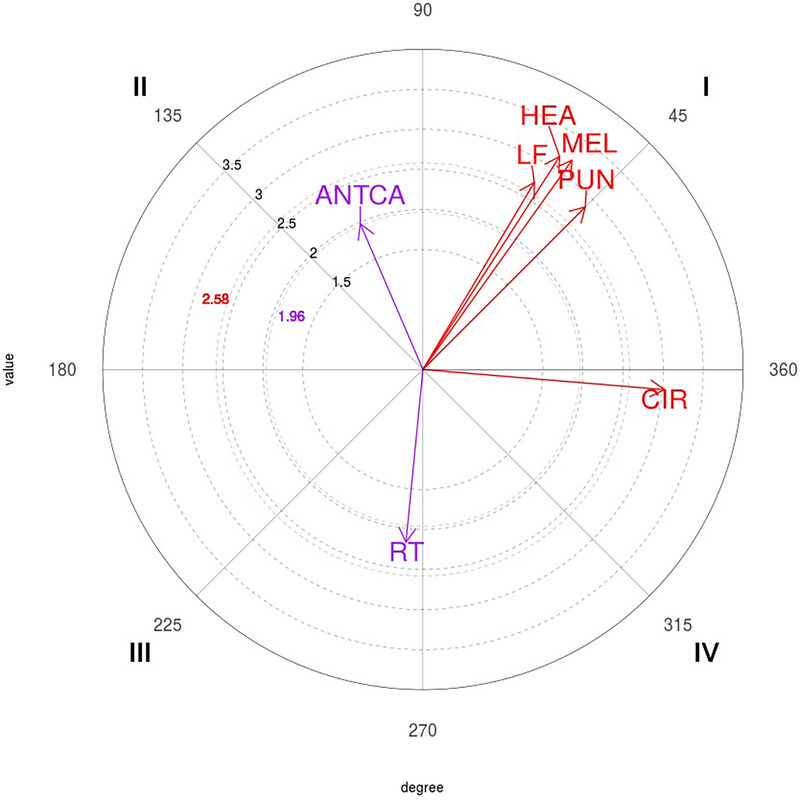
Representation of the behavioral map for the three-point (3P) focal behavior. LF, left; HEA, head, MEL, melee; PUN, punch; CIR, circular; RT, right; ANTCA, anticipatory counterattack.

The polar coordinate map of the interactions between 3P focal behavior and conditional behaviors is presented in Figure 3.

Supplementary Table S5 shows the results of quadrant I function estimation for 3P focal behavior. The other quadrants (II, III, and IV) do not yield results in any model, as they each contain only one significant relationship in the polar coordinate analysis.

The data show minimal variation, with extremely low relative and absolute errors and an explained variance of 100% (1). The linear, polynomial, exponential, and logarithmic regression models and polynomial interpolation model demonstrated a perfect fit to the data, with a correlation coefficient of −1 between *X* and *Y* across all five models. Notably, polynomial interpolation achieves the lowest error values (10^−16^), indicating the highest accuracy in data fitting.

### 3.4 4P focal behavior

In quadrant I, mutual excitation emerged with DA and RT. In quadrant II, inhibition in prospective perspective and excitation in retrospective perspective emerged with SIMCA. In quadrant III, there was mutual inhibition with LF (Table 8).

**Table 8:** Results of the polar coordinate analysis for the four-point (4P) focal behavior.

Category	Quadrant	ProspP	RetrospP	Radius	Angle	Category
Direct attack (DA)	I	3.46	0.05	0.01	3.46^a^	0.79
Simultaneous counterattack (SIMCA)	II	−0.39	4.96	1.00	4.98^a^	94.46
Right (RT)	I	1.98	1.05	0.47	2.24^a^	27.90
Left (LF)	III	−1.88	−0.97	−0.46	2.12^a^	207.37

^a^ Significant vectors (radius >1.96).

**Figure 4:**
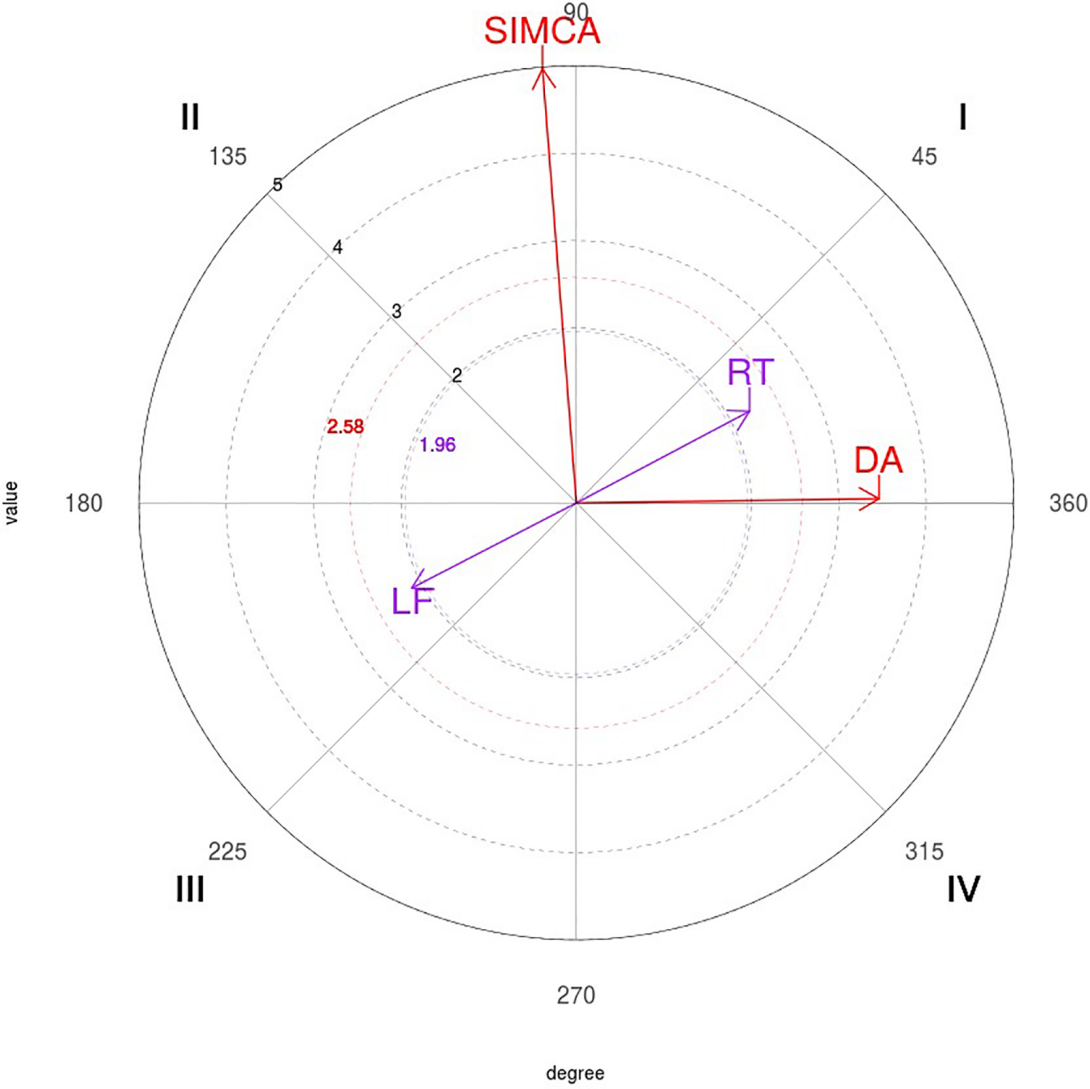
Representation of the behavioral map for the four-point (4P) focal behavior. RT, right; DA, direct attack; LF, left; SIMCA, simultaneous counterattack.

The polar coordinate map of the interactions between 4P focal behavior and conditional behaviors is shown in Figure 4.

The function estimation for 4P focal behavior demonstrates a perfect model fit across all methods (linear, polynomial, exponential, logarithmic, and polynomial interpolation). Quadrant I is the only one exhibiting more than one significant association. The results reveal extremely low errors, with an explained variance of 1 (100%). The correlations between *X* and *Y* (equal to −1) indicate a perfectly negative relationship, while the *Y* vs. *Y* correlation, estimated at 1, confirms the accuracy of the model. Polynomial interpolation achieves the lowest error values (10^−16^), further reinforcing the quality of the fit (Supplementary Table S6).

### 3.5 5P focal behavior

In quadrant I, the behavior exhibited mutual excitation with DA and CIR. In quadrant II, significant relationships were observed with SIMCA and turning (TUR). In quadrant III, mutual inhibition emerged with clashes (CLA) and LIN, while in quadrant IV, a notable association was identified with PUN (Table 9).

**Table 9:** Results of the polar coordinate analysis for the five-point (5P) focal behavior.

Category	Quadrant	ProspP	RetrospP	Radius	Angle
Direct attack (DA)	I	2.32	0.05	2.32^a^	1.17
Simultaneous counterattack (SIMCA)	II	−0.39	2.30	2.34^a^	99.53
Clash (CLA)	III	−1.45	−1.46	2.06^a^	225.15
Linear (LIN)	III	−2.74	−2.76	3.89^a^	225.13
Circular (CIR)	I	2.46	4.04	4.73^a^	58.66
Turning (TUR)	II	−0.30	3.19	3.21^a^	95.34
Punch (PUN)	IV	3.43	−0.53	3.47^a^	351.14

^a^ Significant vectors (radius >1.96).

**Figure 5:**
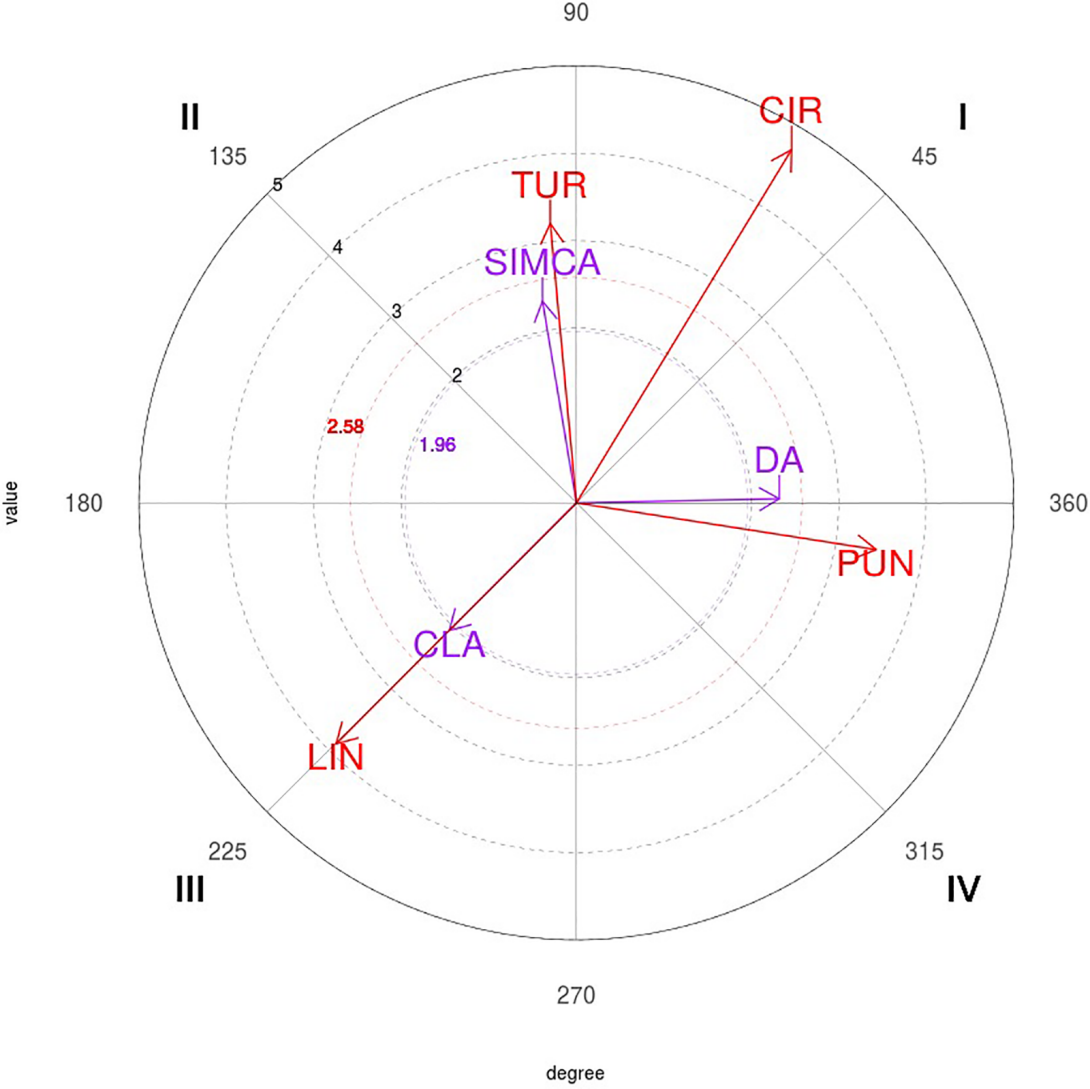
Representation of the behavioral map for the five-point (5P) focal behavior. CIR, circular; DA, direct attack; PUN, punch; CLA, clash; LIN, linear; SIMCA, simultaneous counterattack; TUR, turning.

The polar coordinate map of the interactions between 5P focal behavior and conditional behaviors is shown in Figure 5.

In quadrant I, the function estimation results show a very good fit in all models (linear, polynomial, exponential, logarithmic, and polynomial interpolation). The explained variance and correlation coefficients reach 100% (1). Errors are very low, especially polynomial interpolation achieves an absolute and relative error in the order of 10^−15^ (Supplementary Table S7).

In quadrant II, all four models (linear, polynomial, exponential, and polynomial interpolation) fit the data perfectly, with correlation coefficient and explained variances of 100%. Polynomial interpolation demonstrates a perfect correlation and explained variance (1), and the null values of absolute and relative errors indicate that the model accurately predicts true values (no proportional deviation) (Supplementary Table S8).

In quadrant III, all three functions (linear, polynomial, and interpolation) have an excellent fit, with perfect correlations and explained variances of 100% (1) and errors close to zero, on the order of 10^−15^. The nearly perfect fit of the three functions indicates that the inhibition of actions in this quadrant follows a very predictable pattern (Supplementary Table S9).

## 4 Discussion

The aim of the present study was to examine the technical-tactical scoring actions in the male and female matches at the Roma 2019 WGPS-1 finals using polar coordinate analysis and function estimation. Specifically, polar coordinate analysis was performed to identify the most effective behavioral patterns to score one, two, three, four, and five points. In parallel, function estimation was conducted to validate the data obtained in the polar coordinate analysis and provide a predictive model of the unobserved match data. Considering that taekwondo is a sport that frequently changes rules, it is essential to design tools that contribute to the gradual creation of a stable system and technology that allows each technical gesture to be visualized and analyzed at any time.

The analysis of 1P focal behavior revealed the retrospective and prospective activation of the conditioned behaviors DA, CORR, SUBCA, CIR, SING, and DOU. That is, 1P was achieved as a consequence of a correction or direct attack and a subsequent counterattack (or posterior), preceded or followed by a circular, simple, or double technique. Recently, Gamero-Castillero et al. (3) analyzed the same sample as in our study and applied the same type of analysis to identify the technical-tactical actions with which winning and losing taekwondo athletes managed to score in the finals of the Roma 2019 WGPS-1. Among the winners, scoring a point was preceded and followed by DA and CORR behaviors, with a SING or DOU technique, using a CIR kick to the HEA. Among the losers, scoring a point was preceded and followed by a SUBCA, with a CIR kick executed with the RT leg. The current results contrast with those of previous studies, in which 1P focal behavior activated the ANTCA (6, 41). Although the latter is a relevant tactical action in taekwondo, it was not decisive in scoring a point at the Roma 2019 WGPS finals. In contrast, the relationship between scoring a point and a DA with CIR techniques has been previously observed, indicating that it is frequently used in competitions (11, 14, 16, 41), even though the scoring system was different than the one used in this competition. In parallel, 1P focal behavior inhibited the conditional behaviors IA and LIN in quadrant III, indicating that these actions do not occur for this purpose (8). Regarding the behaviors that are inhibited while achieving 1P actions, previous studies found that among winners, these include IAs, SIMCAs, BLOs, CLAs, and LINs, whereas no inhibited behaviors were observed among the losers at the Roma 2019 WGPS finals, presumably reflecting the more active attacking style of the winners (3). In contrast, during the Rio 2016 OG, the relationships did not show any inhibited behaviors for 1P actions for the winners and only for techniques performed with the back leg for the losers (14). Similarly, during the London 2012 OG (41) 1P actions inhibited some technical-tactical actions, while those at the Rio 2016 OG (13) did not show significant relationships. That is, at the London 2012 OG, openings or ANTCAs with LIN techniques performed with the front LF leg were inhibited behaviors for the women, and openings with the front leg were inhibited behaviors for the men. At the Rio 2016 OG (13), CIR techniques to the HEA were the most inhibited for the women, and techniques performed with the front leg were the most inhibited for the men. Finally, quadrant IV showed that after 1P focal behavior, the conditional behavior SIMCA was prospectively activated. This appears to be a specific characteristic of the losers (3). The results of the function estimation for the focal behavior 1P indicate that tactical relationships, involving DAs, CORRs, and SUBCAs, are critical for success in a match. The analyzed models clearly revealed non-linear interactions, suggesting that tactical sequences must be carefully adjusted to maximize their effectiveness. Additionally, the high consistency of these results indicates that, from a tactical perspective, LINs and IAs are not only ineffective for scoring 1 point but their implementation could also significantly decrease the probability of success.

The analysis of 2P focal behavior revealed the retrospective and prospective activation of the conditioned behaviors SIMCA with a CIR technique to the TRU, and with the LF leg in quadrant I. These conditioned behaviors reflect the most common way of scoring 2P in taekwondo combat, namely executing the CIR kick to the TRU. It is important to highlight that the use of a SIMCA was a new tactical pattern that emerged at the 2019 WGPS, since previous studies (11, 14, 41) showed that the way to score to the TRU was through a DA or ANTCA. This relationship between 2P and kicks to the TRU with a CIR technique has also been previously found (3) among winners, while among losers, the relationship (regarding how to obtain one point) was through a PUN combined with a penalty (GJ) against the opponent. In quadrant II, 2P focal behavior activated the conditional behavior PUN and inhibited it after it was reached. Regarding this relationship, it can be speculated that 1P and 2P actions are complementary (appear at the same time) on multiple occasions since SIMCA also appeared in quadrant IV of the 1P actions. This appears to be a specific characteristic of the winners (3). In parallel, 2P focal behavior inhibited the conditioned behaviors NEU, NOT, and NOA in quadrant III, showing that 2P are obtained in a clear way. Finally, in quadrant IV, the 2P focal behavior prospectively activated the conditional behaviors BLO and LIN techniques, which hypothetically can be explained by an automatic attempt of blocking the technique that has already scored and trying to score immediately after with a LIN technique, as the scoring 2P occurs when a valid technical action is performed at the TRU. The function estimation models for 2P focal behavior indicate that SIMCA, CIR, TRU, and LF are key behaviors that facilitate continued point scoring when successfully executed. In tactical combat analysis, these relationships are crucial for developing dynamic strategies that enable athletes to maximize their scoring actions.

The analysis of 3P focal behavior revealed the retrospective and prospective activation of the conditioned behaviors MEL, PUN, HEA, and LF in quadrant I. This action is highly coordinated because the athlete needs to move to a close distance (melee) for a punch, while at the same time performing a technique to the head with the left side of the body. Previous studies (11, 41) have also found this relationship (partly because the rules established that 3P are obtained through leg techniques to the head), wherein 3P were obtained after cuts (11), blocks (41), and subsequent (or posterior) counterattacks (41). In quadrant II, 3Pfocal behavior retrospectively activated the conditioned behavior ANTCA but subsequently inhibited it. This relationship seems to be especially relevant in winners (3), highlighting the importance of this technical-tactical action for obtaining three points, something that has not been reported in previous studies that analyzed the London 2012 or Rio 2016 OGs (10, 11, 14, 25, 41). The range of possibilities that the winners of the Roma 2019 WGPS-1 display to obtain 3P is noteworthy, as they do so either with a MEL action or an ANTCA, with the LF leg to the HEA, that can include (or not) a PUN. At this point, we would like to highlight the study of Gutiérrez-Santiago et al. (16) that analyzed the <68 kg male category in the 2013 World Taekwondo Championships. They found that the first, second, and fourth most relevant patterns to score were counterattacks. Of these, the second pattern was a counterattack against the initial displacement of the opponent (an anticipated action), and Gamero-Castillero et al. (3) found that the relationship between an anticipatory action and 3P was significant among winners. This is in line with the results of Falcó et al. (6) who found that anticipatory actions were the most difficult tactical actions to be performed, as the recent literature shows, since winners are the ones who are able to perform them successfully (3, 16). In quadrant III, 3P focal behavior inhibited the conditional behavior RT leg, while in quadrant IV, CIR techniques consistently appeared as conditioned behavior. It is interesting to notice that 3P actions were obtained with the left leg (typically the non-dominant leg), while the right leg (usually the dominant leg) was inhibited. Thus, one can expect attempts to score 3P with the LF leg, but not with the RT leg. The function estimation results for 3P focal behavior show perfectly explained variances and correlations between *X* and *Y* across all models. Quadrant I suggests that as the frequency or intensity of a given tactic increases, its effectiveness likely decreases due to opponent adaptation. However, the perfect correlation indicates that MEL, PUN, HEA, and LF function together to execute a valid head-kicking attack. Furthermore, polynomial interpolation suggests that these tactics have a stable and highly predictable pattern, making them useful for developing an effective strategy.

The 4P focal behavior activated the conditional behavior DA with the RT leg both retrospectively and prospectively in quadrant I. In quadrant II, 4P focal behavior retrospectively activated the conditioned behavior SIMCA but subsequently inhibited it. Overall, these results highlight the importance of a SIMCA when the opponents’ performance level is particularly high (6, 10). We expected to find a significant relationship in actions such as CORR, DOU, or TUR technique since 4P can be obtained through a valid TUR kick to the TRU or achieving a CORR to the TRU only. We speculate that the techniques to obtain 4P are very versatile, so only a tactical action performed with the (usually) dominant leg achieves the points. At the same time, it is interesting to highlight that 4P focal behavior inhibited the conditional behavior LF leg in quadrant III, which can be seen as complementary to the way the athletes obtained 3P (as this was with the left leg, while the right leg was inhibited). Previous studies have also shown this complementarity among winners (6), or when analyzing the best athletes (11). They showed a tactical scheme in which one leg was used to score a specific point, while the other was used to obtain another number of points (6, 11). Function estimation models for 4P focal behavior indicate that significant associations with DA and RT are highly effective in generating points during a match. Regression models suggest that these relationships are highly predictable and offer a clear tactical advantage when applied appropriately. From a retrospective perspective, this suggests that before executing a technique valued at 4P, DAs predominantly performed from the RT side are commonly observed. Prospectively, the results show that athletes tend to continue employing DAs and techniques from the RT side after successfully performing a 4P action. This prospective behavior may reflect a clear tactical strategy, exploiting previous success by consistently repeating effective tactical actions.

The 5P focal behavior retrospectively and prospectively activated the conditional behaviors DA and CIR in quadrant I. In quadrant II, it retrospectively activated the conditioned behaviors TUR and SIMCA but subsequently inhibited them. We can interpret this as follows: a DA with a CIR technique is performed by one athlete, and at the same time, a SIMCA with a TUR technique is performed by the other athlete and this obtains the latter 5P. In quadrant III, 5P focal behavior inhibited the conditioned behaviors CLA and LIN techniques, two actions that could stop one obtaining 5P through a defensive action by attempting to impede the opponent's attack using the legs or by pushing the hip (in the back area) of the opponent when performing the TUR technique. In quadrant IV, it prospectively activated the PUN, something that can be seen as a way to close the distance or try to obtain 1P, since after a TUR technique to the HEA, both athletes are quite close. It is important to highlight that, for a punch action to be validated as a point, it must be executed with sufficient force against the electronic body protector, a requirement that is particularly challenging for judges to evaluate (41) and that can come to the scoring table with a delay (explaining why a significant relationship for punches did not appear in 1P scoring). Notably, scoring actions worth four and five points are exclusively performed by winners (3). Previous studies (11, 14, 25, 41) have also observed that not all weight categories, for both men and women, have all possible variations in their scoring repertoire. Finally, function estimation models for the 5P focal behavior indicate that this score is associated with highly difficult or complex techniques. The numerical results clearly reinforce the relationship found through polar coordinate analysis. Both confirm a consistent bidirectional (prospective/retrospective) nature between 5P actions for direct attack with a circular technique and a SIMCA with a TUR technique. The inhibitory relationship reinforces that after executing a SIMCA, the probability of a 5P technique immediately occurring decreases. In contrast, the retrospective excitatory relationship implies that the SIMCA acts as a defensive/counterattack mechanism that breaks the opponent's attack sequence, preventing them from successfully scoring.

### 4.1 Limitations and future directions

Although our study offers new information about high-level taekwondo athletes and their technical-tactical behaviors, it is necessary to acknowledge some limitations that should translate into future developments. First, the application of polar coordinate analysis and function estimation to competitions after the Tokyo 2020 OG should focus on the possible changes from round to round as the most important and recent rule change directly affected the time structure of the match. Second, it would be appropriate to increase the number of match samples in order to stratify the analyses by round of competition, as athletes compete multiple times during the day of competition. Third, the analyses should also include matches of athletes in the cadet and junior age categories since the number of competitions dedicated to these specific populations has increased significantly in recent years. Finally, it would be interesting to compare the technical-tactical behaviors between winning and losing athletes to identify the patterns that lead to success in competition.

### 4.2 Practical applications

The results of the present study have a direct impact on practice as the information provided by polar coordinate analysis and function estimation allows coaches and physical trainers to easily study and understand the possible technical-tactical behaviors of high-level athletes (11–15). The reported information allows for the generation of new training and competition strategies, considering the technical-tactical developments resulting from rule changes (4). In this sense, it is important to highlight that when the rules are stable for longer periods (there are no substantial changes), the technical-tactical repertoire of the athletes increases, and to score, athletes have a clear pattern for each of them. In addition, the interpretation of function estimation can be specifically used to develop targeted training plans that optimize the use of these techniques and tactics based on their likelihood of success in scoring points (23). Therefore, coaches can either utilize the results obtained from this competition to replicate the same strategy during their matches, since the athletes were the best in the competition, or to develop other strategies to nullify the opponent's strategy. To that end, to replicate the strategy, it is recommended to use quadrants I and II, while to develop new strategies, quadrant III may be of help.

## 5 Conclusion

The application of polar coordinate analysis and function estimation to the finals of the Roma 2019 WGPS-1 allowed us to define the different tactical actions that establish a significant pattern. Thus, one point was obtained through a direct attack, a correction, or a subsequent counterattack; two points were obtained through a simultaneous counterattack; three points were obtained through an anticipation or a melee tactical action, while four points and five points were obtained through a direct attack or a simultaneous counterattack. The other tactical actions, such as an indirect attack, clash, block, or dodge, did not show a significant relationship in the strategy adopted by the taekwondo athletes. Furthermore, two points and three points were obtained with the left leg, while four points were obtained with the right leg while three and four points inhibited the right and left side of the body, respectively, highlighting the importance of the actions performed using one side of the body or the other. Linear techniques are also inhibited when obtaining one and five points. Additionally, it is important to emphasize the excitation of the conditioned circular behavior in the one-, two-, and five-point focal behaviors before or after scoring, as there was a high probability of executing a circular kick, but a significant relationship was not observed for linear techniques, showing that the circular technique was the most used.

## Data Availability

The raw data supporting the conclusions of this article will be made available by the authors, without undue reservation.
