# Supplementary material for: Which behavioral patterns score points in taekwondo matches? An analysis of the Roma 2019 World Grand Prix finalists
Source: Front Sports Act Living. 2025 Apr 14;7:1572945. doi: 10.3389/fspor.2025.1572945 (PMC12034700; doi:10.3389/fspor.2025.1572945)
Supplement: Supplementary file 1 [file Table1.docx]

**Supplementary Table S1.** Estimation of quadrant III functions for the one-point (1P) focal behavior.

| **Formal definition** | **Approximation Method** | **Absolute Error** | **Relative Error** | **Correlation between X and Y** | **Explained Variance between X and Y** | | **Correlation between Estimated X and Y** | | **Explained Variance between Estimated X and Y** | | **Correlation Estimated Y vs Y** | | **Explained variance Estimated Y vs Y** | **Function color** | **Graphic** |
| --- | --- | --- | --- | --- | --- | --- | --- | --- | --- | --- | --- | --- | --- | --- | --- |
| f(x) = -2.901 + x * -1.335 | Linear regression | 1.3739E-14 | 6.8695E-15 | -1 | 1 | -1 | | 1 | | 1 | | 1 | | Black | 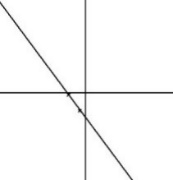 |
| f(x) = (0.938 * (x^2)) + (1.130 * (x^1)) + (-1.720) | Polynomial regression | 1.3739E-14 | 6.8695E-15 | -1 | 1 | -1 | | 1 | | 1 | | 1 | | Red | 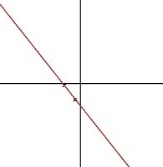 |
| f(x) = -0.23 - 1.336 * (x + 2) | Polynomial interpolation | 5.32907E-15 | 2.66454E-15 | -1 | 1 | -1 | | 1 | | 1 | | 1 | | Brown | 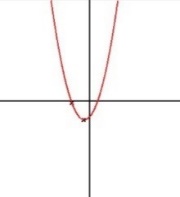 |
| 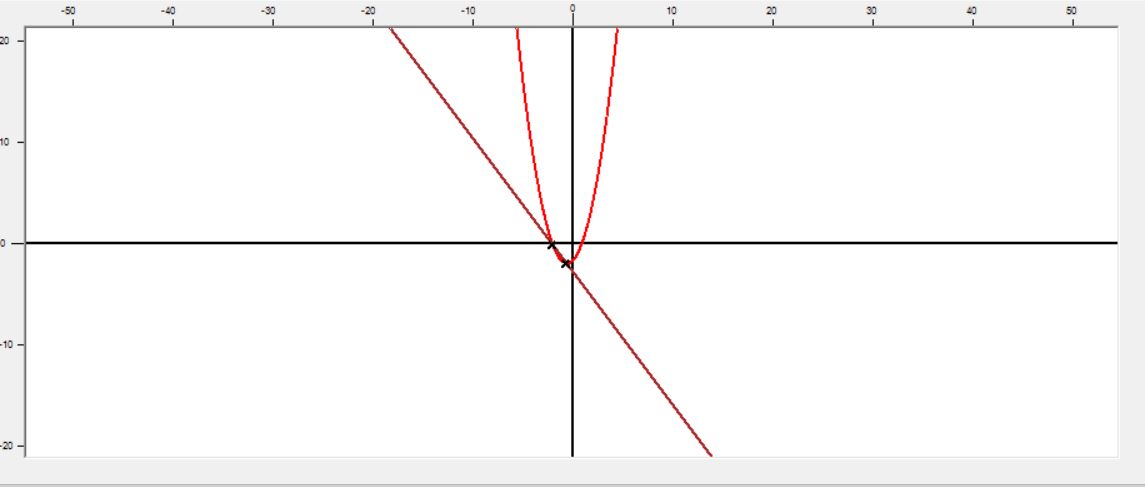 | | | | | | | | | | | | | | | |

**Supplementary Table S2.** Estimation of quadrant I functions for the two-point (2P) focal behavior.

| **Formal definition** | **Approximation Method** | **Absolute Error** | **Relative Error** | **Correlation between X and Y** | **Explained Variance between X and Y** | **Correlation between Estimated X and Y** | **Explained Variance between Estimated X and Y** | **Correlation Estimated Y vs Y** | **Explained variance Estimated Y vs Y** | **Function color** | **Graphic** |  |
| --- | --- | --- | --- | --- | --- | --- | --- | --- | --- | --- | --- | --- |
| f(x) = 3.215 + x * -1.277 | Linear regression | 0.7259 | 0.1814 | -0.9706 | 0.9421 | -1 | 1 | 0.9706 | 0.9421 | Black | 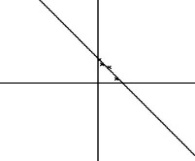 |  |
| f(x) = (-0.464 * (x^2)) + (-0.297 * (x^1)) + (2.928) | Polynomial regression | 0.5571 | 0.1392 | -0.9706 | 0.9421 | -0.9856 | 0.9715 | 0.9847 | 0.9697 | Red | 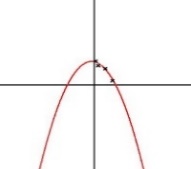 |  |
| f(x) = 3.891 * (0.413^x) | Exponential regression | 1.3746 | 0.3436 | -0.9706 | 0.9421 | -0.9804 | 0.9611 | 0.9229 | 0.8518 | Blue | 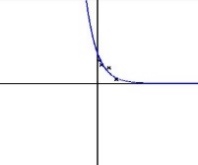 |  |
| f(x) = -0.855 * Log(x) + (1.639) | Logarithmic regression | 1.3785 | 0.3446 | -0.9706 | 0.9421 | -0.9518 | 0.9060 | 0.8988 | 0.8078 | Green | 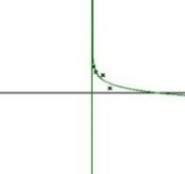 |  |
| f(x) = 3.02 - 2.037 * (x - 0.16) + 1.538 * (x - 0.16) * (x - 0.43) - 1.393… | Polynomial interpolation | 4.77E-15 | 1.19E-15 | -0.971 | 0.9421 | -0.9706 | 0.9421 | 1 | 1 | Brown | 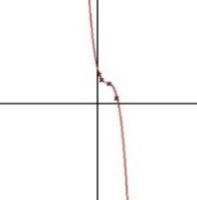 |  |
| 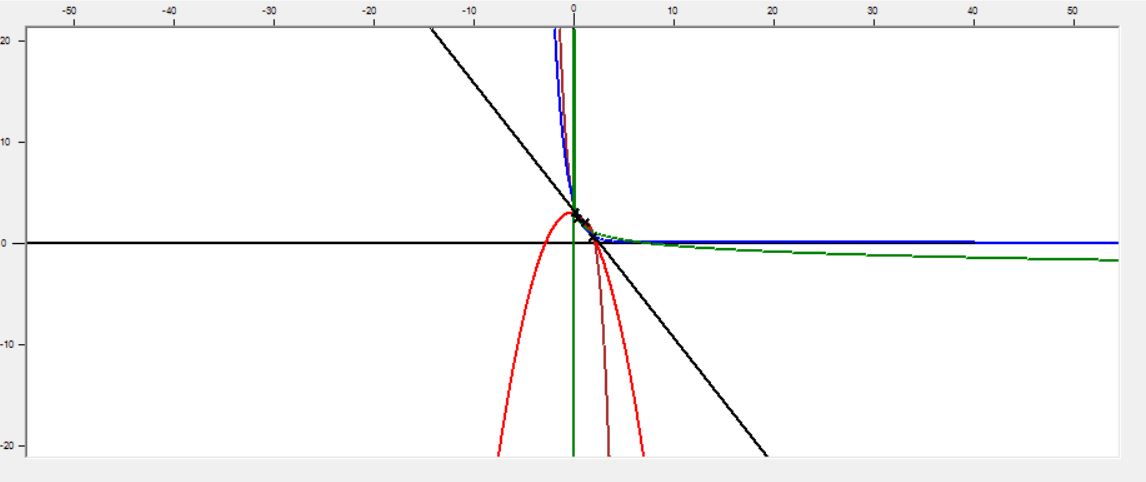 | | | | | | | | | | | | |

**Supplementary Table S3.** Estimation of quadrant III functions for the two-point (2P) focal behavior.

| **Formal definition** | **Approximation Method** | **Absolute Error** | **Relative Error** | **Correlation between X and Y** | **Explained Variance between X and Y** | **Correlation between Estimated X and Y** | **Explained Variance between Estimated X and Y** | **Correlation Estimated Y vs Y** | **Explained variance Estimated Y vs Y** | **Function color** | **Graphic** |
| --- | --- | --- | --- | --- | --- | --- | --- | --- | --- | --- | --- |
| f(x) = -1.866 + x * 0.771 | Linear regression | 0.0076 | 0.0025 | 0.9987 | 0.9974 | 1 | 1 | 0.9987 | 0.9974 | Black | 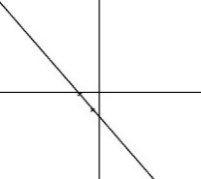 |
| f(x) = (-0.891 * (x^2)) + (-0.617 * (x^1)) + (-2.401) | Polynomial regression | 3.02E-14 | 1.01E-14 | 0.9987 | 0.9974 | 0.9987 | 0.9974 | 1 | 1 | Red | 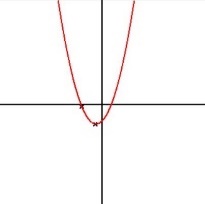 |
| f(x) = -2.53 + 0.818* (x + 0.86) - 0.891 * (x + 0.86) * (x + 0.75) | Polynomial interpolation | 4.44E-16 | 1.48E-16 | 0.9987 | 0.9974 | 0.9987 | 0.9974 | 1 | 1 | Brown | 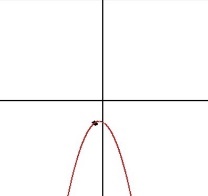 |
| 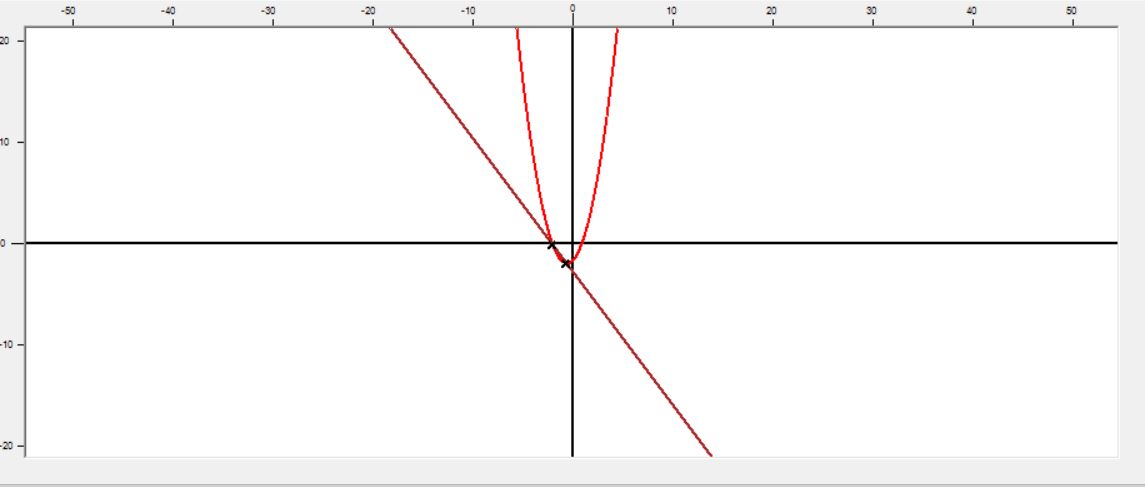 | | | | | | | | | | | |

**Supplementary Table S4.** Estimation of quadrant IV functions for the two-point (2P) focal behavior.

| **Formal definition** | **Approximation Method** | **Absolute Error** | **Relative Error** | **Correlation between X and Y** | **Explained Variance between X and Y** | **Correlation between Estimated X and Y** | **Explained Variance between Estimated X and Y** | **Correlation Estimated Y vs Y** | **Explained variance Estimated Y vs Y** | **Function color** | **Graphic** |
| --- | --- | --- | --- | --- | --- | --- | --- | --- | --- | --- | --- |
| f(x) = -2.263 + x * 0.333 | Linear regression | 5.9952E-15 | 2.9976E-15 | 1 | 1 | 1 | 1 | 1 | 1 | Black | 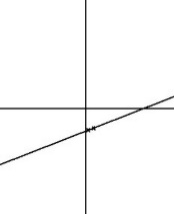 |
| f(x) = (0.375 * (x^2)) + (-0.124 * (x^1)) + (-2.165) | Polynomial regression | 7.3274E-15 | 3.6637E-15 | 1 | 1 | 1 | 1 | 1 | 1 | Red | 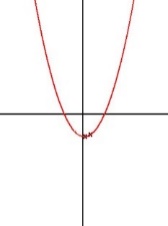 |
| f(x) = 0.182 * Log(x) + (-1.939) | Logarithmic regression | 8.8817E-16 | 4.4408E-16 | 1 | 1 | 1 | 1 | 1 | 1 | Green | 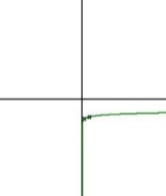 |
| f(x) = -2.17 + 0.333 * (x - 0.28) | Polynomial interpolation | 2.2204E-16 | 1.1102E-16 | 1 | 1 | 1 | 1 | 1 | 1 | Brown | 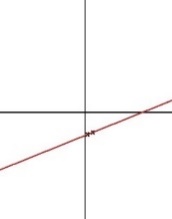 |
| 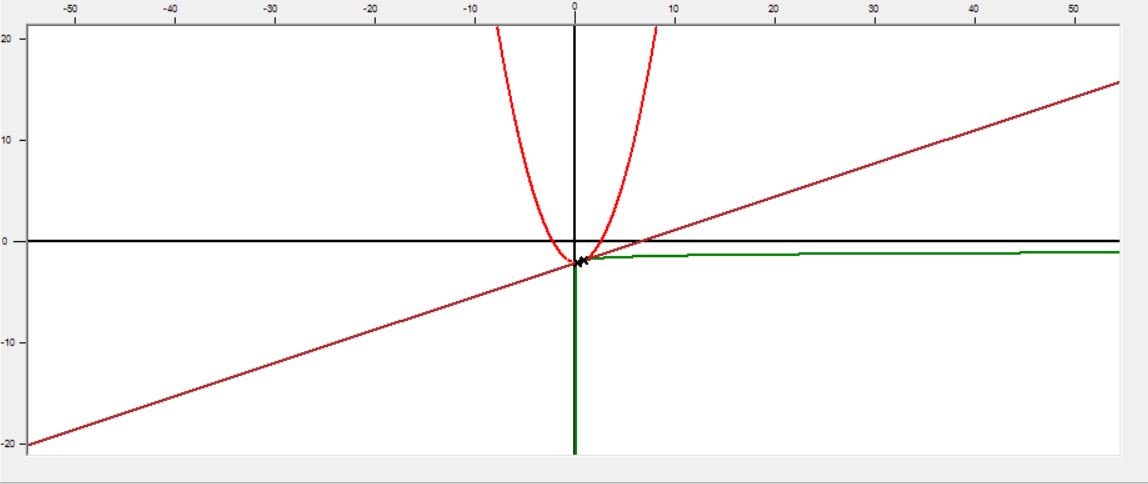 | | | | | | | | | | | |

**Supplementary Table S5.** Estimation of quadrant I functions for the three-point (3P) focal behavior.

| **Formal definition** | **Approximation Method** | **Absolute Error** | **Relative Error** | **Correlation between X and Y** | **Explained Variance between X and Y** | **Correlation between Estimated X and Y** | **Explained Variance between Estimated X and Y** | **Correlation Estimated Y vs Y** | **Explained variance Estimated Y vs Y** | **Function color** | **Graphic** |
| --- | --- | --- | --- | --- | --- | --- | --- | --- | --- | --- | --- |
| f(x) = 2.388 + x * -0.676 | Linear regression | 6.3976E-15 | 3.1988E-15 | -1 | 1 | -1 | 1 | 1 | 1 | Black | 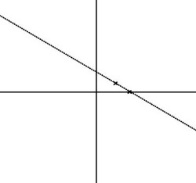 |
| f(x) = (-0.282 * (x^2)) + (0.854 * (x^1)) + (0.461) | Polynomial regression | 2.9282E-15 | 1.4641E-15 | -1 | 1 | -1 | 1 | 1 | 1 | Red | 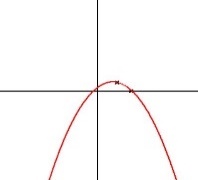 |
| f(x) = 61.674 * (0.128^x) | Exponential regression | 3.747E-16 | 1.8735E-16 | -1 | 1 | -1 | 1 | 1 | 1 | Blue | 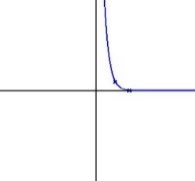 |
| f(x) = -1.792 * Log(x) + (2.274) | Logarithmic regression | 1.7347E-15 | 8.6736E-16 | -1 | 1 | -1 | 1 | 1 | 1 | Green | 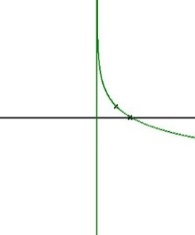 |
| f(x) = 1.05 - 0.675675675675676 * (x - 1.98) | Polynomial interpolation | 4.0245E-16 | 2.0122E-16 | -1 | 1 | -1 | 1 | 1 | 1 | Black | 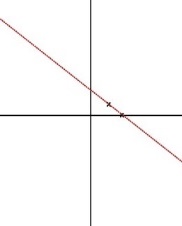 |
| 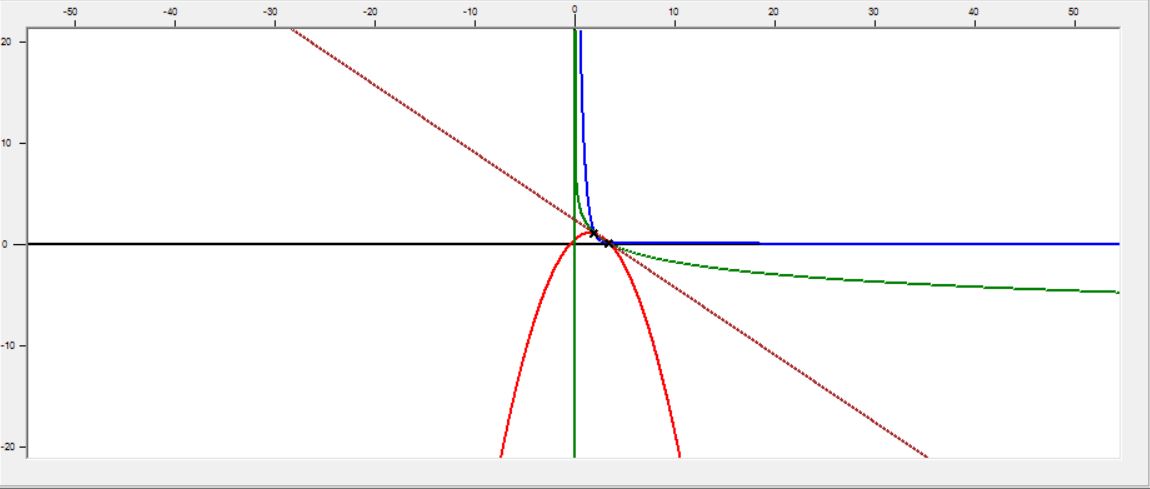 | | | | | | | | | | | |

**Supplementary Table S6.** Estimation of quadrant I functions for the four-point (4P) focal behavior.

| **Formal definition** | **Approximation Method** | **Absolute Error** | **Relative Error** | **Correlation between X and Y** | **Explained Variance between X and Y** | **Correlation between Estimated X and Y** | **Explained Variance between Estimated X and Y** | **Correlation Estimated Y vs Y** | **Explained variance Estimated Y vs Y** | **Function color** | **Graphic** |
| --- | --- | --- | --- | --- | --- | --- | --- | --- | --- | --- | --- |
| f(x) = 2.388 + x * -0.676 | Linear regression | 6.3976E-15 | 3.1988E-15 | -1 | 1 | -1 | 1 | 1 | 1 | Black | 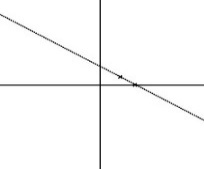 |
| f(x) = (-0.281 * (x^2)) + (0.854 * (x^1)) + (0.461) | Polynomial regression | 2.9282E-15 | 1.4641E-15 | -1 | 1 | -1 | 1 | 1 | 1 | Red | 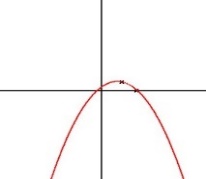 |
| f(x) = 61.674 * (0.128^x) | Exponential regression | 3.747E-16 | 1.873E-16 | -1 | 1 | -1 | 1 | 1 | 1 | Blue | 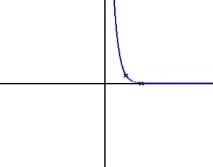 |
| f(x) = -1.792 * Log(x) + (2.274) | Logarithmic regression | 1.7347E-15 | 8.6736E-16 | -1 | 1 | -1 | 1 | 1 | 1 | Green | 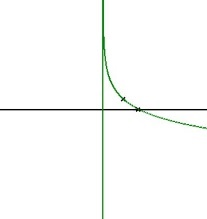 |
| f(x) = 1.05 - 0.676 * (x - 1.98) | Polynomial interpolation | 4.0245E-16 | 2.0122E-16 | -1 | 1 | -1 | 1 | 1 | 1 | Brown | 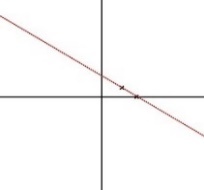 |
| 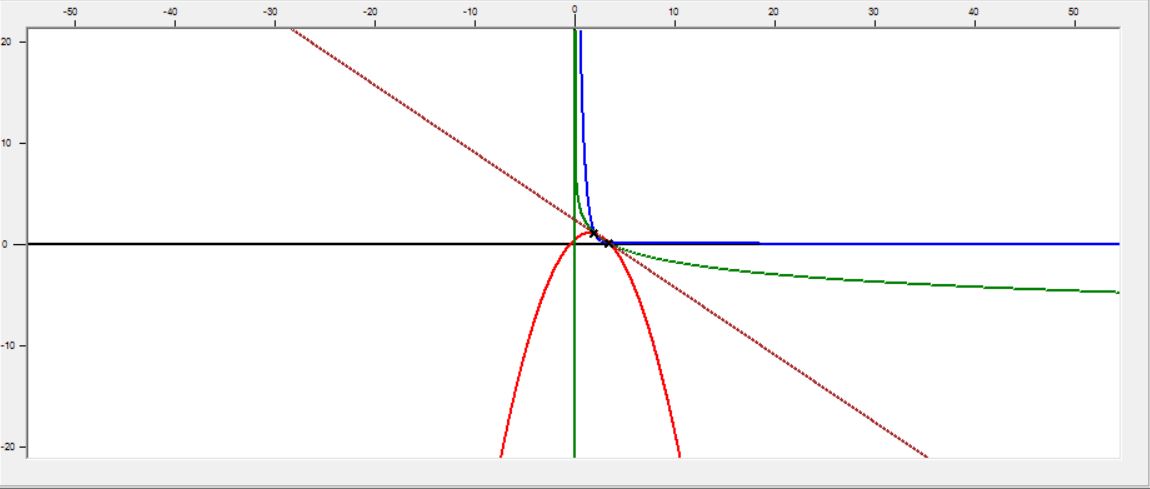 | | | | | | | | | | | |

**Supplementary Table S7.** Estimation of quadrant I functions for the five-point (5P) focal behavior.

| **Formal definition** | **Approximation Method** | **Absolute Error** | **Relative Error** | **Correlation between X and Y** | **Explained Variance between X and Y** | **Correlation between Estimated X and Y** | **Explained Variance between Estimated X and Y** | **Correlation Estimated Y vs Y** | **Explained variance Estimated Y vs Y** | **Function color** | **Graphic** |
| --- | --- | --- | --- | --- | --- | --- | --- | --- | --- | --- | --- |
| f(x) = -66.070 + x * 28.500 | Linear regression | 8.00957E-13 | 4.00478E-13 | 1 | 1 | 1 | 1 | 1 | 1 | Black | 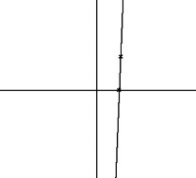 |
| f(x) = (6.966 * (x^2)) + (-4.796 * (x^1)) + (-26.316) | Polynomial regression | 2.50494E-14 | 1.25247E-14 | 1 | 1 | 1 | 1 | 1 | 1 | Red | 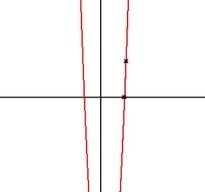 |
| f(x) = 1.232E-33 * (4211.8^x) | Exponential regression | 1.87032E-12 | 9.35158E-13 | 1 | 1 | 1 | 1 | 1 | 1 | Blue | 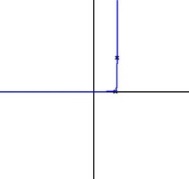 |
| f(x) = 68.096 * Log(x) + (-57.257) | Logarithmic regression | 8.11975E-13 | 4.05988E-13 | 1 | 1 | 1 | 1 | 1 | 1 | Green | 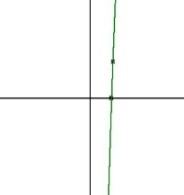 |
| f(x) = 0.05 + 28.5 * (x - 2.32) | Polynomial interpolation | 3.55271E-15 | 1.77636E-15 | 1 | 1 | 1 | 1 | 1 | 1 | Brown | 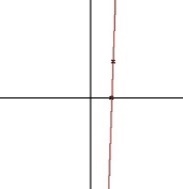 |
| 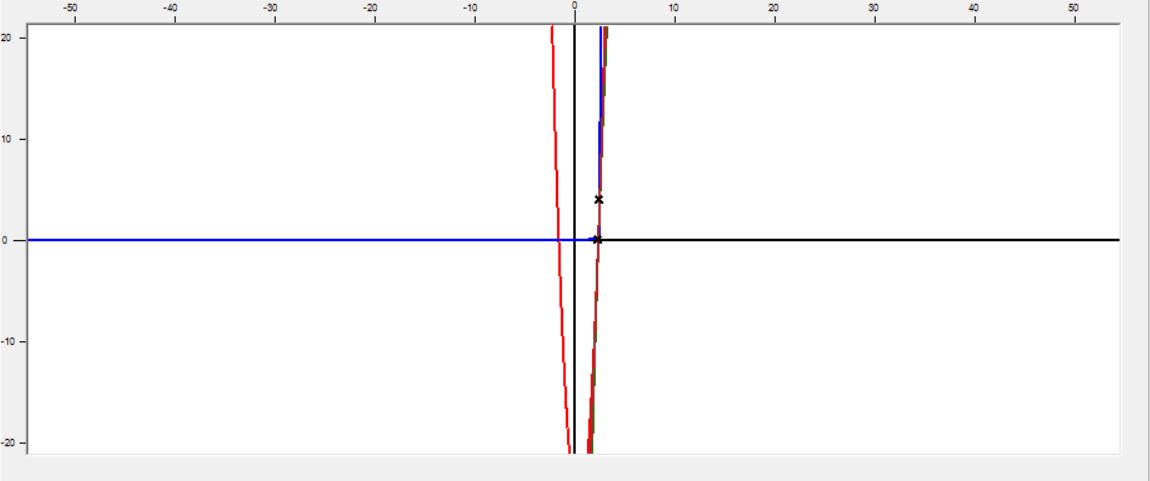 | | | | | | | | | | | |

**Supplementary Table S8.** Estimation of quadrant II functions for the five-point (5P) focal behavior.

| **Formal definition** | **Approximation Method** | **Absolute Error** | **Relative Error** | **Correlation between X and Y** | **Explained Variance between X and Y** | **Correlation between Estimated X and Y** | **Explained Variance between Estimated X and Y** | **Correlation Estimated Y vs Y** | **Explained variance Estimated Y vs Y** | **Function color** | **Graphic** |  |
| --- | --- | --- | --- | --- | --- | --- | --- | --- | --- | --- | --- | --- |
| f(x) = 6.156 + x * 9.889 | Linear regression | 8.8817E-15 | 4.4408E-15 | 1 | 1 | 1 | 1 | 1 | 1 | Black | 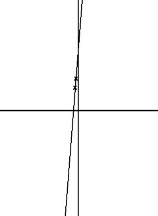 |  |
| f(x) = (-10.667 * (x^2)) + (2.529 * (x^1)) + (4.909) | Polynomial regression | 1.5543E-14 | 7.7715E-15 | 1 | 1 | 1 | 1 | 1 | 1 | Red | 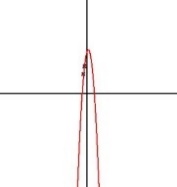 |  |
| f(x) = 9.491 * (37.886^x) | Exponential regression | 8.8817E-15 | 4.4408E-15 | 1 | 1 | 1 | 1 | 1 | 1 | Blue | 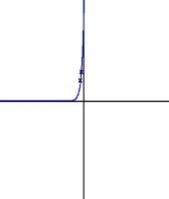 |  |
| f(x) = 2.3 + 9.889 * (x + 0.39) | Polynomial interpolation | 0 | 0 | 1 | 1 | 1 | 1 | 1 | 1 | Brown | 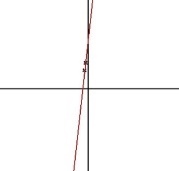 |  |
| 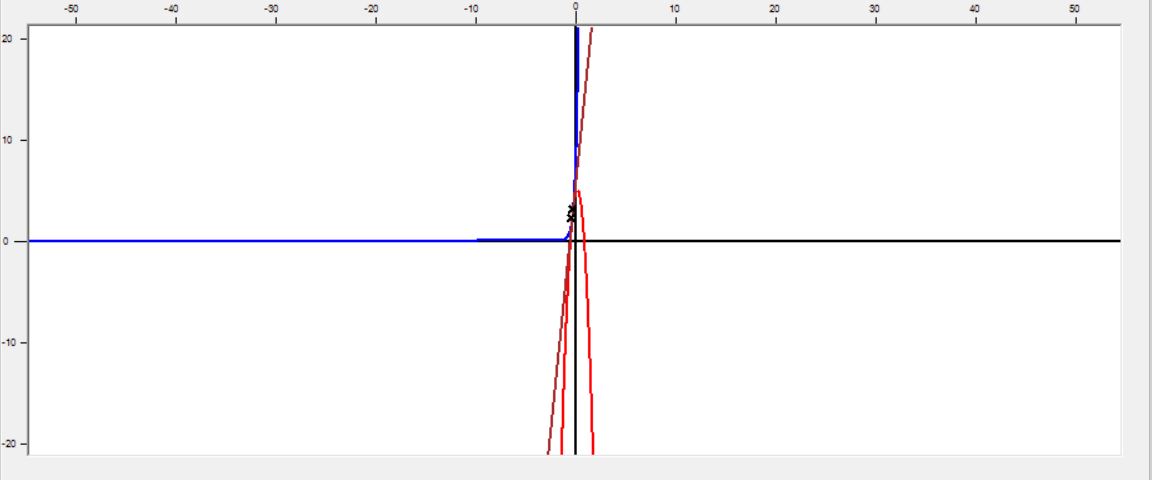 | | | | | | | | | | | | |

**Supplementary Table S9.** Estimation of quadrant III functions for the five-point (5P) focal behavior.

| **Formal definition** | **Approximation Method** | **Absolute Error** | **Relative Error** | **Correlation between X and Y** | **Explained Variance between X and Y** | **Correlation between Estimated X and Y** | **Explained Variance between Estimated X and Y** | **Correlation Estimated Y vs Y** | **Explained variance Estimated Y vs Y** | **Function color** | **Graphic** |  |
| --- | --- | --- | --- | --- | --- | --- | --- | --- | --- | --- | --- | --- |
| f(x) = 0.001 + x * 1.008 | Linear regression | 5.10703E-15 | 2.55351E-15 | 1 | 1 | 1 | 1 | 1 | 1 | Black | 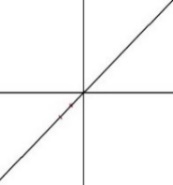 |  |
| f(x) = (-0.323* (x^2)) + (-0.345* (x^1)) + (-1.282) | Polynomial regression | 5.9952E-15 | 2.9976E-15 | 1 | 1 | 1 | 1 | 1 | 1 | Red | 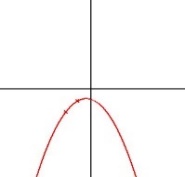 |  |
| f(x) = -2.76 + 1.008 * (x + 2.74) | Polynomial interpolation | 5.32907E-15 | 2.66454E-15 | 1 | 1 | 1 | 1 | 1 | 1 | Brown | 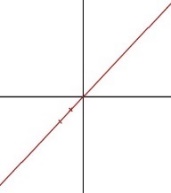 |  |
| 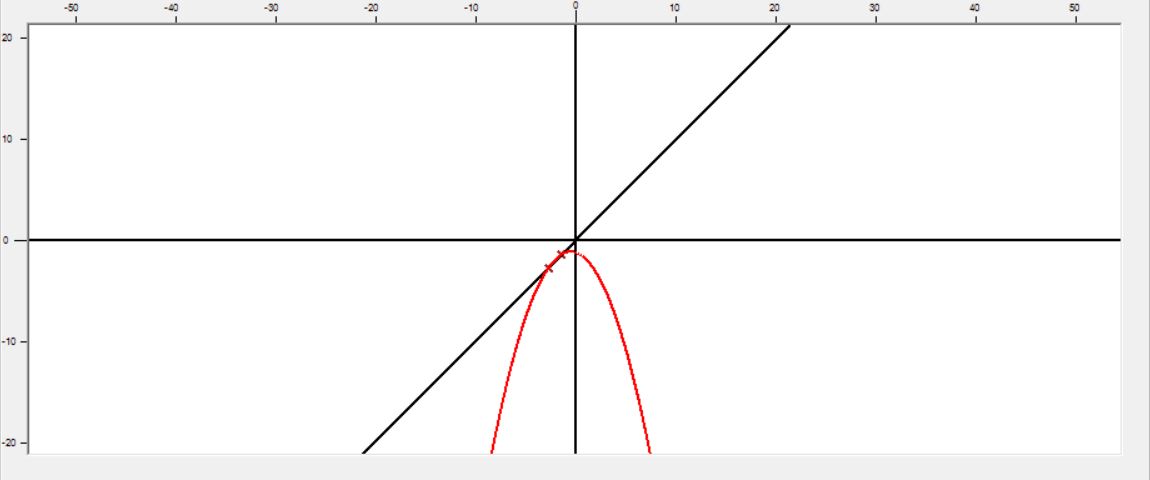 | | | | | | | | | | | | |
